# Really Asymptomatic? Health-Related Quality of Life and Objective Clinical Foot Characteristics among 5–10-Year-Old Children with a Flexible FlatFoot

**DOI:** 10.3390/jcm12093331

**Published:** 2023-05-07

**Authors:** Saidas Žukauskas, Vidmantas Barauskas, Ramunė Degliūtė-Muller, Emilis Čekanauskas

**Affiliations:** Department of Paediatric Surgery, Lithuanian University of Health Sciences, 44307 Kaunas, Lithuania; vidmantas.barauskas@kaunoklinikos.lt (V.B.); ramune.degliute@kaunoklinikos.lt (R.D.-M.); emilis.cekanauskas@kaunoklinikos.lt (E.Č.)

**Keywords:** foot posture index, paediatric flexible flatfoot, footprints, quality of life

## Abstract

The potential effects of asymptomatic flexible flatfoot (FF) on children’s health-related quality of life (QoL) and objective clinical foot characteristics have been poorly investigated in the literature. Therefore, this study aimed to analyse these indicators, comparing the children with asymptomatic FF and a control group. Methods: In total, 351 children were enrolled in this cross-sectional study—160 children with asymptomatic FF and 191 controls (children with normal feet). The children and their parents completed the Paediatric Quality of Life Inventory (PedsQL^TM^ 4.0). The objective foot characteristics included clinical foot posture measures, footprints, general hyperlaxity, and X-ray measurements. Results: Children with asymptomatic FF had a significantly lower QoL (overall and all four dimensions). The parents’ assessment of the QoL of their children with asymptomatic FF in most cases was lower compared to their children’s self-reported QoL. Moreover, almost all clinical foot measures also had significantly worse profiles among asymptomatic FF cases compared to the controls. This was observed with the Foot Posture Index-6 (FPI-6), the navicular drop (ND) test, the Chippaux–Smirak Index (CSI), Staheli’s Index (SI), the Beighton scale, and radiological angles (except the talo-first metatarsal angle). Conclusion: The findings suggest that asymptomatic FF not always reflects a normal foot development. This condition is related to decreased health-related quality of life, so the 5–10-year-old children’s and their parents’ complaints should be considered more closely in identification, treatment, and monitoring plans.

## 1. Introduction

Paediatric flexible flatfoot is usually described as a foot posture with a deficiency in, or the insufficiency of, a longitudinal arch in the midfoot [1,2]. It is regarded as an anatomical variation in the foot structure [3], and clinicians have simply divided this foot posture into painless (asymptomatic flexible flatfoot) and painful (symptomatic flexible flatfoot) [4,5,6,7,8]. This was due to the fact that paediatric flexible flatfoot follows a history of natural improvement over time [8,9] and also because it is easier to reach an agreement for treatment in a child with a symptomatic flexible flatfoot [10].

Nevertheless, the abnormal foot shape often becomes a cause for concern for parents who may then choose to seek medical referral for their child [11,12,13]. Parents have many questions for the orthopaedic specialist related to the child’s quality of life, physical activity, and the possible consequences of the existing foot deformity in the child’s future [8,14,15,16]. Even healthcare specialists are often concerned about the child’s foot flatness and their concern often turns into a frequent referral to an podiatrist [8,17]. At this point, for the clinical practitioner, in the decision-making process, it is essential to distinguish between the different forms of flatfoot—asymptomatic or symptomatic flexible flatfoot. If the child expresses pain in the feet, and the parents also have concerns about the shape of the child’s feet, this commonly used combination opens up the possibility of using an understandable and approved treatment algorithm for flexible symptomatic flatfoot [1,4,18,19,20,21,22,23]. On the other hand, if the child does not express or note pain in the feet, but only the parents are worried about the child’s flat feet, then clinicians should alleviate their parents’ worry and explain that children are born with flexible flatfoot which naturally develops the normal arch in the first decade of life or remains asymptomatic [24].

The debate between the treatment and observation of asymptomatic flexible flatfoot is still highly contested [4]. While the primary goal of treating symptomatic flatfoot is a relief of pain by adjusting the parameters of the foot to a normal foot, asymptomatic flexible flatfoot is targeted to the prevention of future disability, as well as the avoidance of significant symptoms and the impairment of health-related QoL [25,26,27]. Thus far, there are little data available to explain why a flexible flatfoot either remains asymptomatic or becomes painful [11].

Currently, methods for assessing flat feet in children rely on a combination of age and foot posture [28]. While there are many studies that compare the arches of healthy children’s feet to those with flattened or absent arches, there is a lack of clinical evidence linking these objective indicators of foot structure and function to health-related quality of life (QoL) in children with only asymptomatic flexible flat feet. It is important to understand the impact of flat feet on children’s health-related quality of life, as this could inform clinical decision making and potentially improve outcomes. Additionally, assessing and treating flat feet in children may be important for preventing or addressing any potential musculoskeletal issues that could arise in the future. Therefore, more research is needed to better understand the relationship between objective indicators of foot structure and function, as well as health-related quality of life in children with asymptomatic flexible flat feet. It is a known fact that most children’s foot arches progress quickly and are formed by the age of five. With this in mind, children entered this study between the ages of five and ten. To date, it is quite hard to find studies focused on a specific evaluation of differences between children with normal arches and those with asymptomatic FF in terms of health-related QoL and objective clinical foot characteristics, including the FPI-6, the ND test, the CSI, SI, calcaneal pitch angle (Tc), talo-first metatarsal angle (Tm), and talocalcaneal angle (Tc). The potential effect of asymptomatic FF on children’s health-related QoL and objective clinical foot characteristics at different ages has been poorly investigated in the literature. The aim of this study was to analyse these indicators by comparing the children with asymptomatic FF to a control group.

The results of this study could be very important for clinicians in making informed judgments about the health-related QoL of painless and flexible flatfoot children compared to children with normal arches of the same age and gender. This study is one of the few to investigate child and parent reports of health-related QoL related to asymptomatic FF and its effects on clinical indicators (FPI-6 and ND test scores), footprint parameters (the CSI and SI), hypermobility (the Beighton scale), and radiological parameters (Tm, Cp, and Tc) in 5-to-10-year-old children with asymptomatic FF and non-flatfoot children.

## 2. Materials and Methods

### 2.1. Sample and Procedure

The subjects who participated in this cross-sectional study were divided into children with asymptomatic FF and without flexible flatfeet between the ages of 5 and 10. The data were obtained from 351 children in the period of 2019 and 2022. Subjects with asymptomatic FF (defined as positive following a tip-toe test (Jack test)) [28] and an FPI-6 score equal to and more than 6 were included, as well as healthy feet children with an FPI-6 score between 0 and 5 without any joint deformities. Subjects were excluded if they had any trauma in a year prior to the study, painful feet, or neurological and congenital conditions (such as cerebral palsy and genetic disorders, rigid flatfeet, etc.); if they had leg and feet surgeries; if they already used orthoses or inlets; or if they disagreed to be involved in the study.

Information on age, sex, body weight, height, and underlying diseases was collected. The sample was well balanced regarding gender and age, with a slight over-representation of a 5-year-old group and with less in the 9- and 10-year-old groups. Table 1 shows the demographic and anthropometric profile of the study participants.

According our research protocol, each child’s foot posture of both feet was evaluated by the FPI-6 [18]. Six and more scores on asymptomatic FF were collected and compared with those defined as the controls.

**Figure 1:**
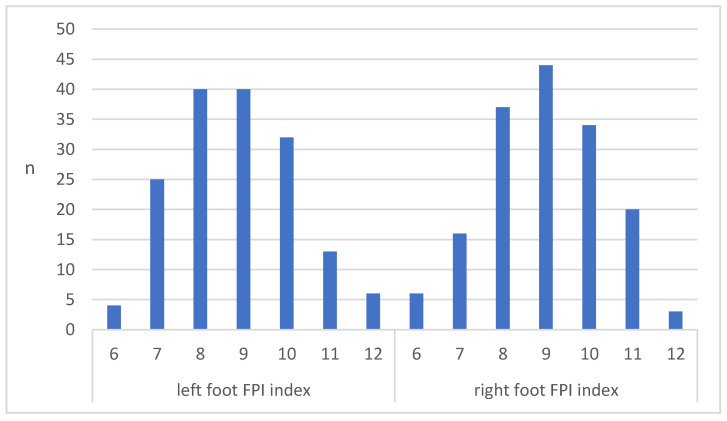
Objective assessment of asymptomatic flexible flatfoot: the FPI-6 (*n* = 160).

Figure 1 shows the distribution of asymptomatic FF, according to the FPI-6 profile of the participants and their split into left-foot and right-foot groups.

All procedures and investigations performed in the present study were in accordance with the ethical standards of the Institutional and National Research Committee and with the 1964 Declaration of Helsinki and its later amendments and comparable ethical standards (BE-2-2). All parents and/or legal guardians of the participating children signed a written informed consent statement before the participants were brought into the study.

### 2.2. Measures

At the initial assessment after the screening protocol, all participants included in the study underwent the same clinical orthopaedic, quality of life, footprint, and radiographic evaluations.

All patients had the same exact clinical examination, which was recorded on a standardised document sheet made for frequent usage in orthopaedic evaluation. In this research, we looked at several sets of parameters that may be used to identify cases of flexible flatfoot.

The following indicators were assessed:

The navicular drop test [29,30], which is used in addition to the foot posture index to evaluate foot shape. A navicular discrepancy >9 mm affirms a pronated foot type [29,30]. By comparing the navicular height with the foot in a weight-bearing and non-weight-bearing neutral position, the navicular drop may be determined. A navicular drop score higher than 9 mm suggests a pronated foot type, and a score between 5 and 9 mm shows a neutral foot type.

Footprints of left and right feet [31], which are used to calculate Chippaux–Smirak (CSI) and Staheli (SI) indexes [32]. Distribution to categories: CSI 0.1–29.9%—normal arch, CSI > 45%—flatfoot [33]; SI 0.44–0.89—normal arch, SI > 0.89—flatfoot [33]. While seated in a chair, patients were able to place both feet on Harris and Beath footprint mats to conduct the footprint-based examination. After rising up with an equal weight on both feet and sitting back down, footprint data were taken, and any evidence of foot movement or imbalance was discarded. The CSI evaluates the relationship between the shortest midfoot length and the longest metatarsal heads, whereas the SI evaluates the corresponding relationship between the lowest midfoot length and the longest heel.

Right- and left-foot radiographs, which are used in the standard weight-bearing foot. Lateral view angles collected are as follows: talo-1st metatarsal angle (Tm), calcaneal pitch angle (Cp), and talo-calcaneal angle (Tc) [34]. Distributions to the flatfoot category are as follows: Tc > 45°, Tm > 4°, and Cp < 20° [18,35]. The indication for an X-ray examination in symptom-free children was set as a measure for research purposes to exclude the potential foot pathology related to bone structures.

A Beighton hypermobility score was counted for joint ligamentous laxity assessment. Values range from 0 to 9 [19,36], with >4 indicating general joint ligamentous laxity [19,37].

A Paediatric Quality of Life Inventory TM Generic Core Scale 4.0 (PedsQL™ 4.0) questionnaire was used for participants (children and parents) [38,39]. The questionnaire is used for healthy and diseased inhabitants [38,40]. Scores from 0 to 100 are given, with higher scores indicating better functioning [40,41]. Through the use of focus groups and cognitive interviews, the PedsQL^TM^ 4.0 Generic Core Scale assessment tool was developed to gauge health-related QoL in kids aged 5–7 and 8–12, as well as other ages. There are a total of 23 questions on the questionnaires, and they include topics such as physical health (8 questions), mental health (5 questions), social health (5 questions), and academic success (5 questions). Both the child and parent were given their own questionnaires to fill out; the PedsQL^TM^ 4.0 was filled out by the child, while the proxy questionnaire equivalents were filled out by the parent. A questionnaire for 5–7-year-olds was used to evaluate the smiley system, since children of this age cannot write. Parents read the question and were shown three smileys with the values 0, 2, and 4. This is provided in the PedsQL™ 4.0 questionnaire methodology [38,42]. More than 50 percent of the questions must be answered in order for the answers to be evaluated. It was explained that the subjects have the right not to answer the questions if they do not understand them.

### 2.3. Statistical Analysis

The data were analysed in SPSS for Windows (version 27). The results with a *p* value of <0.05 were considered to be statistically significant. Differences between the two groups, i.e., children with asymptomatic FF and controls, were examined with the independent *t*-test, taking Levene’s test into account for the equality of variances. To compare the differences between variables with a various range of possible values, the effect size was used (Cohen’s d).

The children’s and parents’ assessments were compared using the paired sample t-test. They were compared using the linear regression model for the potential prediction of FPI-6 scores with an adjustment for age and gender. The standardised beta coefficient was the main indicator of variable strength in the models.

## 3. Results

Children and their parents completed the quality-of-life questionnaires. Statistical analysis showed that both children with asymptomatic FF and their parents tended to rate the quality of life as poorer compared to the controls in all quality-of-life domains (Table 2). Comparing the different domains of quality of life, the most expressed differences were in the physical domain (delta values of 2.37 and 5.22) and then in the emotional domain (delta values of 1.80 and 3.84). All differences were highly significant (*p* < 0.001).

Furthermore, the children’s and parents’ assessments of the children’s quality of life were compared. It was found that in cases of asymptomatic FF, the parents consistently tended to rate their children’s quality of life as lower than the children themselves. The total difference in quality of life between children and parents was 29.0 pts, and the domain’s differences were very similar (ranging from 28.4 to 32.0 pts). In contrast, the controls faced much less expressed differences between the children’s and parents’ perceptions of quality of life. Here, differences in the total score reached only 3.0 pts, which was mostly due to largest and significant differences in the school domain. All of this suggests that the parental perception of children’s health-related quality of life is essentially affected by asymptomatic FF as a condition.

After discovering that children’s and parents’ assessments of quality of life significantly differ, we built a model to see whether either of these two assessments show a stronger relationship to an objective measure of flatfoot—known as the FPI-6. This was conducted using a linear regression model with adjustments for age and gender. The findings reveal that a parental assessment of children’s quality of life is much stronger than children’s self-reports (Table 3).

Comparing the objective indicators, the majority of indicators showed significant differences between cases with asymptomatic FF and controls (*p* < 0.001). The most expressed differences were observed with CSI (delta 6.74 and 6.81), and were slightly lower with the FPI-6 (delta values of 6.36 and 6.61) and the ND test (delta values of 6.07 and 6.24). Among X-ray measurements, the calcaneal pitch angle had the largest differences between the study groups, while the talocalcaneal angle was the only objective measure that was not different between the study groups (Table 4).

## 4. Discussion

Our study results highlight the need for a revision of the current best practises involving children only with asymptomatic FF at specific ages from five to ten years. As we noticed, a child with asymptomatic FF receiving significantly lower total PedsQL^TM^ 4.0 scores in physical, emotional, social, and school activities may suggest they have a symptomatic flatfoot posture. In another study, Kothari et al. [43] also confirmed differences in health-related QoL in children with FF, mostly in the physical block. However, an inclusion bias of participants with mixed symptomatic and asymptomatic characteristics was given. With the same issue in biomechanical studies [15,44,45], in three-dimensional gait analysis involving neutral flat symptomatic and flat asymptomatic subjects, lower physical scoring and slower walking speed were also noticed. Moreover, kinematic and kinetic changes in planes with FF and normal-shaped feet were affirmed in children [46,47].

Despite these abnormal findings in children’s QoL, parents also evaluated their children’s health-related QoL in our study. The results were found to be even worse than the children’s self-reported results. This may be explained by the parents’ worries about their children’s future in connection with pain in lower limbs, possible foot deformities, and tibialis posterior tendon disfunction [48,49,50,51,52]. Furthermore, if the painless FF becomes symptomatic and is expressed in the perception of pain, the health-related QoL of children is significantly affected and leads to conservative or surgery treatment options [53,54]. Parents’ concerns about the child’s flatfoot posture causes them to make frequent visits to ambulatory clinics for a podiatric consultation [8]. They most often encounter an answer when the childhood condition of the foot is normal [55] and when no treatment is urgent, especially when it is painless. In this study, we observed and argued against this “normal” condition to the clinicians; typical feet children’s parents had a much higher overall score in their children QoL than asymptomatic FF parents. This fact justifies parents’ concerns about their asymptomatic FF children.

Diagnosing FF and its subsequent categorisation into asymptomatic and symptomatic FF is a truly daunting task [56]. First, the podiatrist is at a crossroad in terms of the natural history of FF and pathological FF. In a prospective study, Martínez-Nova 2018 et al. [9] showed five-to-eleven-year-old children’s feet transformation to neutral position from highly pronated and pronated conditions, according to FPI-6 scores. Second, Camurcu 2021 et al. [14] noticed that physicians who started paediatric FF treatment with orthoses had no significant difference of QoL between subjects; on the contrary, parents had significantly lower scores when children were using orthoses. Third, another important deducing factor is the age of the child, as well as the child’s ability to express his or her grievances compared to adults. This may raise doubts for the orthopaedics in treating this asymptomatic FF. Even so, if an abnormal arch persists and bone deformity continues, this might seriously affect a child’s health-related QoL [56]. Dabholkar 2020 et al.’s study on flat-footed adults aged between 20 and 40 years highlighted that QoL is affected. This impacts the adult’s quality of life in the form of foot pain, difficulty to conduct daily living tasks (such as walking on uneven ground, walking fast, running, and maintaining balance), and the ability to find comfortable footwear [57]. Moreover, in their study with 1798 adults, Almutairi 2021 et al. concluded that flatfoot is associated with acute and chronic back pain [58]. The Framingham Foot Study [59], which used adults over 50, argued that flatfoot posture was associated with increased odds of hammer toes and overlapping toes, and also increased in pronated foot function. They underscored the utility of clinical input in understanding the relations between foot posture, function, and foot disorders.

Due to the lack of clarity of the boundary between asymptomatic FF and symptomatic FF, the physician, i.e., the paediatric foot orthopaedic surgeon, has developed an unambiguous approach in his daily practice to the classification of children’s feet according to pain alone [25,43]. Clinical practice and clinical practice guidelines do not hesitate to define algorithms for the treatment of flexible flatfoot in children, i.e., treatment is only required when the child expresses foot pain or points to sore areas in the feet [4,5,12,37]. It is recommended that conservative treatment should start with physiotherapy, orthopaedic footwear, and the encouragement of more active movement. Moreover, if the child’s expressed or reported foot pain persists, and if the parents also insist on the persistence of the child’s foot pain, surgical foot treatment is recommended [11,23,60]. Again, the main indicator of the success of the surgery is the disappearance of, or reduction in, the child’s foot pain, as well as the satisfaction expressed by the parents in noting the shaped foot arches. On the other hand, there is a sort of hesitation regarding the success of surgical treatment in children with FF. This is because the desired result is not always obtained, according to the understanding of the surgeons and the parents. The disappearance of pain, even after a precise surgical intervention, is not always achieved by assessing the child’s health-related QoL [6,17,43]. This means we need to go back to health-related QoL and to look earlier than when the pain appears for the child, so that there is no doubt that the treatment algorithm has started.

The second approach to this study was based on static FF foot indicators on the relationship between the parents’ and the children’s overall health-related QoL. The objective FF parameters (clinical FPI-6; the ND test; CSI footprint; SI; and Tm, Cp, and Tc radiographs), which directly signal the paediatric asymptomatic FF form, also show a sensitive distribution in this asymptomatic FF group. We hypothesised that parents are overly sensitive to their children’s health-related QoL in relation to objective indicators used in the scientific literature. Unfortunately, as the indicators towards the FF shape of the children’s foot changed, health-related QoL also deteriorated. Finally, most studies have highlighted the frequent use of FPI-6 as a sensitive, specific, and predictive tool in evaluating paediatric asymptomatic and symptomatic FF. This is very important for both researchers and clinicians in daily practice [61,62]. We compared this routine tool, FPI-6, with the health-related QoL of parents and children to see whether there are changes based on age and gender. So, no clinically significant differences were found.

This study addressed the gap in the literature regarding asymptomatic FF children; health-related QoL; and valid, reliable, and daily usable FF parameters. Understanding the asymptomatic FF in developing children and its impact to health-related QoL helps to detect any persisting deviations beyond a certain stage of development. This allows more information to be obtained on how asymptomatic FF children can choose the right treatment option and avoid atypical dynamic future changes in the lower limbs. Studies that compare paediatric asymptomatic FF and normal feet, as well as their influence on health-related QoL, are hugely lacking. Differences on the sample characteristics and measurement procedures, e.g., not having an accurate FPI-6 or other select parameters, make it difficult to compare the results of different studies. This is because an evaluation of the child’s foot with a FPI-6 indicator to a score of 6 or 12 score makes a lot of sense. As the foot complex is nearly developed in adolescence, there is a need for normative baseline data in this population which can help to compare deviations in asymptomatic FF children with health-related QoL.

Finally, this study is one of the few in this field that attempts to answer questions raised in the paediatric orthopaedic community on children with painless FF. First, the classification of paediatric FF into symptomatic and non-symptomatic characteristics is stagnant because our study results show the existing similarities in health-related QoL with children with painful FF; this was also confirmed in Kothari et al.’s study [43]. Second, the “normal” foot shape. We can observe this foot shape at appropriate ages in the children which may change over time given the stages of natural foot development, especially in the first decade of life. However, the case of whether this will change or not at this time may indicate whether lower health-related QoL is associated with developed foot arches, and parents could have reasonable doubts about their children’s feet in terms of QoL. Considering this, the management of paediatric asymptomatic FF may not only consist of observations, but also discussions with parents about possible intervention. Third, the wide range of paediatric foot structural characteristics at baseline and their comparison with health-related QoL could provide further information when interventions such as conservative treatment are applied, ensuring changes in children’s health-related QoL, while monitoring and evaluating changes in objective indicators of deformity.

Our study’s results support this notion by suggesting that early intervention, even with conservative treatment, may lead to improved asymptomatic FF in the future. First, we recommend that healthcare professionals adopt a more proactive approach towards identifying and treating asymptomatic FF in children. This may involve conducting routine screening for FF in infants and young children, as well as educating parents about the importance of early intervention. Conservative treatments, such as stretching and strengthening exercises, and footwear modifications should start before considering more invasive treatments such as surgery. Improvements associated with early intervention are consistent with the findings of previous studies, such as [19], which demonstrated the potential benefits of early intervention in addressing FF in children. Our findings add to this body of literature by demonstrating that even asymptomatic FF may produce benefits from early intervention, which could prevent future complications and improve foot function.

The main limitation of our study was the lack of a separate symptomatic FF group and the inclusion of dynamic parameters (gait analysis), as well as their relations to typical and painless children. Further studies should be performed in prospective design with different sizes to see if these findings are consistent or specific to certain subgroups.

## 5. Conclusions

Children with asymptomatic FF have a significantly lower overall QoL compared to children with normal feet. Parents’ assessment of the health-related QoL of their children with asymptomatic FF is more sensitive. These findings suggest that asymptomatic FF not always reflects a normal foot development. This condition is related with decreased health-related quality of life, so the 5–10-year-old children’s and their parents’ complaints should be considered more closely in identification, treatment, and monitoring plans.

## Figures and Tables

**Table 1:** Characteristics of participants based on age, gender, and study groups.

Characteristic		AFF ^1^		Controls		Total
		Boys	Girls	Boys	Girls	
Age, years	5	20	20	21	20	81
	6	20	20	15	15	70
	7	20	20	15	15	70
	8	20	20	15	15	70
	9	-	-	15	15	30
	10	-	-	15	15	30
Subtotal		80	80	96	95	351
Total		160		191		

^1^ asymptomatic flexible flatfoot.

**Table 2:** Differences and effect sizes between children with and without asymptomatic flexible flatfoot: health-related quality of life.

Quality of Life ^1^		AFFF	Controls	Difference	Cohen’s Delta	t	*p*
Children	total	56.67	81.60	−24.92	−2.38	−21.45	<0.001
	physical	53.84	85.93	−32.09	−2.37	−22.08	<0.001
	emotional	49.90	82.91	−33.00	−1.80	−16.72	<0.001
	social	66.09	84.29	−18.20	−0.98	−8.72	<0.001
	school	60.03	70.65	−10.62	−0.57	−5.34	<0.001
Parents	total	27.62	84.63	−57.01	−5.77	−50.38	<0.001
	physical	21.72	86.14	−64.42	−5.22	−46.04	<0.001
	emotional	25.63	83.09	−57.46	−3.84	−34.58	<0.001
	social	35.28	84.11	−48.83	−3.21	−28.44	<0.001
	school	31.38	84.27	−52.89	−3.76	−33.65	<0.001

^1^ PedsQL 4.0, Paediatric Quality of Life Inventory TM Generic Core Scale 4.0.

**Table 3:** Health-related quality of life in relation to FPI-6 scores following children’s and parents’ assessments in the linear model, adjusted by age and gender.

Foot	Indicator	B	Beta	*p*	R^2^
Left	Age	0.73	0.30	<0.001	0.86
	Gender	−0.47	−0.06	0.007	
	assessed by parents	−0.10	−0.82	<0.001	
	assessed by children	−0.04	−0.17	<0.001	
Right	Age	0.68	0.28	<0.001	0.86
	Gender	−0.23	−0.03	0.179	
	assessed by parents	−0.10	−0.82	<0.001	
	assessed by children	−0.04	−0.18	<0.001	

**Table 4:** Differences and effect sizes between children with and without asymptomatic flexible flatfoot: objective measures.

Indicator	Foot	AFF ^1^	Controls	Difference	Cohen’s Delta	t	*p*
Clinical measurements							
Foot Posture Index-6 (FPI)	Left	8.84	0.57	7.27	6.36	58.81	<0.001
	Right	8.98	0.55	7.32	6.61	61.06	<0.001
Navicular drop test	Left	15.54	1.41	10.31	6.07	53.02	<0.001
	Right	15.57	1.20	10.35	6.24	55.35	<0.001
Footprints							
Chippaux–Smirak index (CSI)	Left	78.74	3.87	40.85	6.81	61.12	<0.001
	Right	78.76	3.82	41.23	6.74	60.66	<0.001
Staheli’s Index	Left	1.36	0.54	0.68	4.10	36.41	<0.001
	Right	1.36	0.52	0.68	4.10	36.33	<0.001
Hyperlaxity							
Beighton scale		4.37	1.71	0.68	0.42	3.53	<0.001
X-ray measurements							
Calcaneal pitch angle	Left	11.56	3.05	−10.70	−3.19	−27.49	<0.001
	Right	11.74	2.96	−10.67	−3.48	−29.53	<0.001
Talocalcaneal angle	Left	70.69	4.37	0.58	0.14	1.16	0.246
	Right	70.58	5.15	0.96	0.19	1.61	0.108
Talo-1st metatarsal angle	Left	12.51	3.53	1.55	2.66	22.54	<0.001
	Right	12.79	3.95	1.60	2.74	23.26	<0.001

^1^ asymptomatic flexible flatfoot.

## Data Availability

The datasets generated and analysed during the current study are not publicly available due to privacy reasons, but are available from the corresponding author on reasonable request.
